# Heritability and Genome-Wide Association Study of Dog Behavioral Phenotypes in a Commercial Breeding Cohort

**DOI:** 10.3390/genes15121611

**Published:** 2024-12-17

**Authors:** Nayan Bhowmik, Shawna R. Cook, Candace Croney, Shanis Barnard, Aynsley C. Romaniuk, Kari J. Ekenstedt

**Affiliations:** 1 Department of Basic Medical Sciences, College of Veterinary Medicine, Purdue University, West Lafayette, IN 47907, USA or drnayandvm11@gmail.com (N.B.); cook311@purdue.edu (S.R.C.); 2 Department of Comparative Pathobiology, College of Veterinary Medicine, Purdue University, West Lafayette, IN 47907, USA; ccroney@purdue.edu (C.C.); barnard4@purdue.edu (S.B.); aromaniu@purdue.edu (A.C.R.)

**Keywords:** inbreeding coefficient, social fear, non-social fear, startle response, genetic risk score

## Abstract

**Background**: Canine behavior plays an important role in the success of the human–dog relationship and the dog’s overall welfare, making selection for behavior a vital part of any breeding program. While behaviors are complex traits determined by gene × environment interactions, genetic selection for desirable behavioral phenotypes remains possible. **Methods**: No genomic association studies of dog behavior to date have been reported on a commercial breeding (CB) cohort; therefore, we utilized dogs from these facilities (*n* = 615 dogs). Behavioral testing followed previously validated protocols, resulting in three phenotypes/variables [social fear (SF), non-social fear (NSF), and startle response (SR)]. Dogs were genotyped on the 710 K Affymetrix Axiom CanineHD SNP array. **Results**: Inbreeding coefficients indicated that dogs from CB facilities are statistically less inbred than dogs originating from other breeding sources. Heritability estimates for behavioral phenotypes ranged from 0.042 ± 0.045 to 0.354 ± 0.111. A genome-wide association analysis identified genetic loci associated with SF, NSF, and SR; genes near many of these loci have been previously associated with behavioral phenotypes in other populations of dogs. Finally, genetic risk scores demonstrated differences between dogs that were more or less fearful in response to test stimuli, suggesting that these behaviors could be subjected to genetic improvement. **Conclusions**: This study confirms several canine genetic behavioral loci identified in previous studies. It also demonstrates that inbreeding coefficients of dogs in CB facilities are typically lower than those in dogs originating from other breeding sources. SF and NSF were more heritable than SR. Risk allele and weighted risk scores suggest that fearful behaviors could be subjected to genetic improvement.

## 1. Introduction

When choosing a dog to join a household, a dog’s behavior and temperament are generally considered along with physical appearance, size, and compatibility with owner lifestyle, among other factors [1]. For example, in a survey of adopters obtaining dogs from five shelters, dog behavior toward people was one of the top three reasons for selecting a particular dog or puppy [2]. Behavioral issues are also one of the main reasons given for the relinquishment of dogs to shelters [3]. However, a study in commercial breeding (CB) facilities indicated that some behavioral traits may be predicted, which may permit some proactive interventions. Specifically, rehomed dogs had lower social fear and non-social fear, and higher trainability in their new homes if they exhibited higher sociability in their original kennel environment [4]. Behavior is also one of several tools used to evaluate an animal’s welfare state. Behavior may provide information on a dog’s affective state, which is a salient component of animal welfare [5]. Additionally, a dog’s behavior may be related to its physiological state both at baseline and in response to stressors, further supporting its effectiveness in evaluating welfare [6]. Taken together, behavior is a vital characteristic for consideration when determining dogs’ potential success in family homes and their overall welfare states whilst residing in kennel environments. Canine behavior is flexible and can be modified, to some extent, across a dog’s lifespan. Several factors, including those that are intrinsic (e.g., genetics (including breed), sex, temperament, and hemispheric specialization) and extrinsic (e.g., early learning environment and exposure to specific stimuli), can influence the development and expression of dog behavior [7].

Inheritance plays a major role in behavior, although genetics rarely account for more than half of behavioral phenotypic variance [8]. Behaviors are genetically complex traits controlled by many genes of small effect, along with the environment and gene × environment interactions. Modern genomic techniques such as single-nucleotide polymorphism (SNP) array genotyping allow the capture of genetic data across all chromosomes, enabling the genetic evaluation of complex traits such as behavioral phenotypes. Once SNP data are generated, it, together with phenotype information, can be evaluated in several ways. Analyses can include the average inbreeding coefficient (IBC) (an approximate measurement of the probability of identity by state of different pairs of genes) [9], estimations of SNP-based heritability (the amount of phenotypic variability attributed to an individual’s genetics, as opposed to the individual’s environment, based on genome-wide SNP data) [10], genome-wide association studies (a survey of all markers for the association between a genomic region and a phenotype, which also enables a query into potential positional candidate genes), and the calculation of genetic risk scores (an aggregate metric for predicting the measure of susceptibility to a given phenotype/disease [11]). To date, all existing genomic association studies on dog behaviors, including fearfulness, aggression, temperament, personality, herding, and trainability traits, and more, have been performed on dogs in homes, without consideration of the breeding source, using C-BARQ or other questionnaire data (i.e., a survey-based canine behavioral evaluation tool) [12,13,14,15,16,17], and never on dogs exclusively from CB facilities. Because dogs from CB kennels are the subject of much ongoing welfare research [18], it would be illuminating to examine the genetics driving behavioral phenotypes in this group. For example, demonstrating that strong genetic selection is possible either for desirable behavioral phenotypes or away from undesirable behavioral phenotypes would aid in CB kennels’ abilities to produce dogs that are more likely than not to behave in ways that meet prospective pet families’ expectations.

Therefore, the current study utilized hundreds of dogs from CB facilities throughout the Midwestern United States. Each dog’s behavioral phenotype was evaluated, and blood collected for DNA genotyping on high-density canine SNP arrays. The hypothesis-free aims were to (1) compare SNP-based IBCs in a cohort of dogs specifically from CB facilities to a breed background cohort of dogs of the same breed, in order to reveal if dogs in CB facilities are typically more or less inbred compared to dogs of the same breed from the general breed background; (2) calculate heritability estimates for three phenotypes (social fear, SF; non-social fear, NSF; and startle response, SR) on a cohort of dogs from CB facilities; (3) carry out genome-wide association analyses for SF, NSF, and SR on a cohort of dogs from CB facilities and scrutinize the genome for positional candidate genes near any suggestive or significant loci; and (4) calculate genetic risk scores for the more extreme ranges of the SF, NSF, and SR phenotypes on a cohort of dogs from CB facilities. Together, these analyses will, for the first time, create a cohesive picture of the inbreeding coefficients and several aspects of the behavioral genetics of dogs originating at CB kennels.

## 2. Materials and Methods

### 2.1. Ethical Statement

The samples and data reported here were generated from a larger study, approved by Purdue University’s Institutional Animal Care and Use Committee (IACUC, #1809001796A006), which focused on the welfare of dogs and puppies raised and bred in CB kennels, and on the behavioral and management factors associated with rehoming outcomes in retiring dogs. All dogs used were owned by private individuals engaged in CB, and owners gave full, informed consent to participate in this study. Low-stress handling techniques were used while performing blood draws to reduce fear and stress. To protect animal and human welfare and safety, if a dog showed signs of extreme fear, behaviors that could potentially harm them or the investigators during testing, or any others indicative of experiencing more than transient distress, the sampling was halted and the dog was not enrolled in the genetic studies.

### 2.2. Animals and Facilities

Forty CB kennels participated from across the Midwestern United States (Indiana, Illinois, Ohio, and Iowa). All kennels were USDA-licensed [19] and met or exceeded federal and state regulations. A total of 664 dogs were enrolled for this study, but only 615 had complete records and were kept for further analysis. A maximum of 20 adult dogs (>18 months of age) were randomly selected (no particular trait drove this selection) among the available kennel population on the first day of the visit. Bitches in the last two weeks of gestation and those nursing puppies were excluded from the study. The average age of all dogs included in the analysis was 3.55 years, ranging from 1 to 10 years old; 82.8% were females (*n* = 509), and 17.2% were males (*n* = 106), representing 50 different purebreds and six mixed breeds dogs (Supplemental Table S1).

#### Breed Background Animals

Previously generated canine SNP data from multiple sources were utilized as a non-CB “breed population background” for the inbreeding coefficient calculations only. Most of these canine SNP data are publicly available from many dozens of previous studies, typically case/control disease-based studies, from around the world. These data have been combined with our private, internal database of additional canine SNP data. Together, this represents SNP data from >10,000 dogs. From this total, only breeds matching those from the CB kennels in the present study, and exceeding the minimum of 10 dogs per breed, were retained. While a small subset of these dogs may have originated at a CB kennel, most of them would not have come from this source; thus, they are used as a breed population background.

### 2.3. Behavioral Assessment

Testing was performed at the CB facilities. Before testing began, dogs were individually confined to the indoor portions of their home pens and given three minutes to habituate to their surroundings. Tests were recorded using a portable camcorder (Sony Handycam HDR-CX405, New York, NY, USA) mounted on a tripod. All dogs underwent a stranger approach test, immediately followed by a reactivity test. These are previously validated tests [20,21] and were performed following the same protocols as described in the original paper.

Briefly, the approach test consisted of three steps during which an unfamiliar person approached the pen door, opened the door, and reached to touch the dog. After each step, the immediate behavioral response of the dog was scored. Next, a treat was offered, and whether the dog ate it or not was recorded. Finally, whether or not the person could touch the dog (touch was scored as “no” if the dog was out of reach or if the dog was in reach but showed overt signs of fear and/or avoidance) was also recorded [22]. The behavioral response of the dogs to each step was scored following the Red–Yellow–Green (RYG) scoring system developed by Bauer et al. [21]. A dog was scored “red (0)” if it showed signs of fear (e.g., flight or freezing), aggression (e.g., growling, teeth baring, or lunging), or stereotypic behavior; “green (2)” if it was undisturbed or showed affiliative behaviors; or “yellow (1)” if it showed an ambivalent approach or could not clearly be scored “red” or “green” [21]. Any signs of aggression during the test were also recorded separately (0 = yes/1 = no).

The reactivity test consisted of 10 steps during which the dog was introduced to a variety of social and non-social stimuli [20]. In brief, the researcher recorded the dogs’ reactions to the following: a rubber mat, a leash, and a plastic cone (these are inanimate novel-objects aimed to assess levels of non-social fear); a life-like statue of a dog (this is often used as a proxy measure of intra-specific sociability); a ball toy and a squeaky toy (these help measure the dog’s playfulness); a simple problem-solving task; an opening umbrella (which was intended to assess startling reaction to a sudden movement); commands of both ‘come’ and ‘sit’ (as indicators of trainability and willingness of the dog to interact with the person); and, finally, a leash looped over the dog’s head (a measure of trainability and reactivity to a direct human interaction). Supplemental Table S2 describes how each step of the test was performed. Dogs’ responses were recorded using a 3-point scale. A dog was scored “0” if she/he showed signs of fear/aggression, or showed no interest in the stimuli; “2” if she/he showed no signs of fear or confident exploration/interaction with the stimuli; or “1” if she/he showed a cautious approach or an ambivalent response to the stimuli [20]. All behavioral assessment tests were performed by three female experimenters trained in the use of the test and its standardization. Inter-rater reliability between these testers was good and is reported elsewhere [20].

### 2.4. Adjustment of Behavioral Data

A subset of dogs from the original study was used for this study, comprising only the dogs for whom a blood draw could be obtained. Behavioral data were analyzed using IBM SPSS (v.28) and SAS v.9.4 (SAS Inst., Cary, NC, USA) software. Following the data management protocols used in previously published work utilizing these tests [4,20], all the behavioral assessment variables (approach test + reactivity test) were consolidated using principal component analysis (PCA, varimax rotation). The PCA extracted four main components explaining 68.5% of the variance (KMO = 0.95) (see Supplemental Table S3): on component one, variables associated with measures of SF had the higher loadings, which included the approach, open, and reach steps of the approach test. The second component had high loading of variables associated with treats and food motivation (FM). Variables associated with measures of NSF had the higher loadings on the third component, including the reaction to the mat, the leash, the cone, and the dog statue. Finally, the reaction to the opening umbrella variables loaded on the fourth component which was a measure of SR. Principal components scores (i.e., standardized scores calculated using the least squares regression procedure in SPSS) were created for each dog and for each extracted component; these were used for further analysis. For the purpose of this study, component two (food motivation) was excluded from further analysis as not relevant to the research questions presented here.

### 2.5. DNA and Genotyping

After the behavioral assessment was completed, whole blood samples were collected via venipuncture (via cephalic vein) and placed into EDTA (anticoagulant) tubes. Blood collection occurred in an area of the kennel where dogs routinely underwent veterinary exams (i.e., quiet room or dogs’ home pens) to ensure a low-stress experience. Dogs were placed on a non-slip surface by a familiar caretaker and were offered baby food (Turkey, Ham, or Chicken and Gravy; Gerber, Nestle, Florham Park, NJ, USA) on a disposable plate or spatula throughout collection. Samples were labeled and stored in a cooler with ice for transport to the Canine Genetics Laboratory (Purdue University, West Lafayette, IN, USA).

DNA was extracted using the Qiagen Puregene DNA extraction kit protocol (QIAGEN N.V., Hilden, Germany). The quality of DNA was verified using a Nanodrop spectrophotometer (Thermo Fisher Scientific, Waltham, MA, USA); DNA was then stored at −80 °C. All DNA samples were genotyped on the Affymetrix Axiom CanineHD SNP Genotyping Array Set (710,000 SNPs) at Affymetrix, Inc. (Santa Clara, CA, USA). Before quality control, 635,984 variants were mapped to the Broad CanFam3.1 genome assembly [23]. Genotypes were phased by SHAPEIT4 [24], followed by imputation for sporadic missing genotypes using IMPUTE2 [25]. Only polymorphic SNPs mapped to autosomes were used in the GWAS analyses (*n* = 493,039), which meant SNPs mapped to mitochondria, X and Y chromosomes, and unmapped in the genome (*n* = 15,962), and monomorphic variants and insertions/deletions (combined, *n* = 126,983) were excluded. Autosomal polymorphic markers were then filtered using MAF > 0.05 and a call rate of >90%, followed by pruning in PLINK [26] using the command --indep-pairwise 200 50 0.6. This quality control process retained a total of 293,519 SNP markers for further analysis (heritability analyses, genome-wide association study, and genetic risk scores).

### 2.6. Inbreeding Coefficient Calculations

PLINK 1.9 [26] was used to merge the raw SNP data of individuals in this study with previously generated SNP data from an internal database (generated for other studies and combined with copious publicly available data) per breed, termed the “breed population background”. Importantly, this database primarily represented dogs from show, pet, and hobby breeders, with only a small minority potentially representing dogs originating from CB facilities. A minimum sample size of 10 dogs for each breed from both the CB dataset and the breed background dataset was required for analysis (*n* = 16 breeds met this criteria); breeds with <10 from either dataset and those without matching breeds were excluded. These sixteen pooled datasets were then subjected to quality control; SNPs with a minor allele frequency of ≤0.05 and a genotyping rate of ≤90% were removed from further analysis. The above quality control analysis was performed by breed, resulting in differing numbers of remaining SNPs for further analysis (least remaining SNPs = Cavalier King Charles Spaniels with 12,406; most remaining SNPs = Australian Shepherds with 172,437; see Table S4, column E “N(NM)”—number of markers used—for each breed). Since each breed has a different set of fixed/nearly fixed SNPs, performing quality control on each breed ensured the removal of those SNPs, avoiding inflation or deflation of IBCs. PLINK [26] was used to identify and prune SNPs in linkage disequilibrium within each breed using the commands --indep-pairwise 200 50 0.6. The --het command was used to calculate the IBC of individuals within each breed. IBCs of dogs from CB facilities were compared to IBCs of same-breed dogs from the breed background population. The F-test of equality of variances was calculated for the IBCs of each pair (by breed) of populations. Next, the appropriate *t*-test was performed depending on if the variances of the two populations were significantly different or not. The *p*-value (*p* < 0.05) for the two-tailed *t*-test was used to determine if the IBCs of the dogs from CB kennels were significantly different from the IBCs observed in a same-breed cohort (breed background) of dogs. It should be noted that these statistics represent within-breed inbreeding and should not be used to compare across breeds.

### 2.7. Preparation of Behavioral Data for Further Genetic Analyses

The three behavioral data components (SF, NSF, and SR), or phenotypes, were adjusted for fixed effects before the genetic association analysis. For example, since the phenotypes were collected from dogs at 40 different kennels, facility might have contributed to variations in the observed phenotypes. Similarly, since both male and female dogs, of different age groups, and representing 50 purebreds and 6 mixed breeds, were used, these factors might also account for variations in the observed phenotypes. Finally, dog breeds vary significantly in body size (mass), which may also influence observed traits. Therefore, all 615 dogs were categorized into five different groups (namely, extra-large (XL), large (L), medium (M), small (S), and extra-small (XS)) based on typical breed size as determined by American Kennel Club breed standards. Next, a variance-standardized genetic relationship matrix was built based on genome-wide SNP data using PLINK [27]. The derived principal components (PCs) with larger variances (i.e., PC1 and PC2) were tested as covariates in the generalized linear model (GLM) in SAS with other fixed effects [such as facility (*n* = 40), dog age as a covariate, sex (*n* = 2), and dog size (*n* = 5)] to control for population structure created by breeds. Behavioral phenotypes were then adjusted using a multiple linear regression model, where phenotypes were considered as the outcome variable and all other significant effects as predictor variables. This was performed using the *lm* function of R in the *STATS* package [28]. The residuals of that fitted model were then treated as the adjusted phenotypes that were used in further analyses.

### 2.8. Heritability Analyses

Heritability is the ratio of additive genetic variance to the total phenotypic variance. Heritabilities were estimated with adjusted behavioral phenotypes and SNP array data using multiple programs, namely, BLUPF90 [29], GEMMA [30], and GCTA [31]. BLUPF90 was performed via the average information REML (AIREML) and Gibbs sampling (100,000 iterations and 20,000 burn-in) methods.

### 2.9. Genome-Wide Association Study

Only dogs with phenotypes available (*n* = 575) were considered in the association analysis. Associations between SNP markers and adjusted behavioral phenotypes were assessed using a univariate linear mixed model with kinship as a covariate in GEMMA v.0.98 [30]. This analysis was performed in two steps. A centered relatedness matrix was calculated in the first step; this matrix was then used in the association analysis as a covariate in the second step to control for kinship (or relatedness). Wald test *p*-values were used to determine significant markers from the association testing. Bonferroni thresholds [*P_bon_* = −log(0.05/number of variants used in the analysis)] were applied to identify significantly associated variants. A suggestive threshold [*P_sug_* = −log(1/number of variants used in the analysis)] was applied if there were no significant variants after the Bonferroni correction. Although approximately one false positive outcome is expected to occur randomly in a complete genome scan with this suggestive threshold, it is still worth reporting [32]. Manhattan and Q-Q plots were generated in R using the *qqman* package [33]. The genomic inflation factor, also known as lambda gc (λgc), was calculated for each association to ensure sufficient correction for population stratification. This inflation factor compares the median chi-squared test statistics to the expected null distribution [34]. The lambda gc was calculated in R [28] using the formula, median (qchisq (1–p, 1))/qchisq (0.5, 1), where p is a vector of the Wald test *p*-values obtained in GWAS analysis.

The protein-coding genes with their gene ontology (GO) terms/definitions (https://geneontology.org (accessed on 2 May 2024)) within a 1 Mb flanking region of each suggestive or significant marker (500 kb up- and downstream) were identified using BioMart Ensembl [35]. However, occasionally, two variants with a distance ≤ 1 Mb formed the same window; in such cases, 500 kb above the SNP with minimum position and 500 kb below the SNP with maximum position created the final genomic regions. The association of identified genes with behavioral phenotypes was investigated using published literature, the UCSC genome browser [36], and VarElect [37].

### 2.10. Genetic Risk Scores

Dogs were grouped into less fearful and more fearful categories based on standard deviations (SDs) above and below the mean adjusted behavioral phenotype in order to compute a genetic risk score (GRS). Thus, dogs with less fearful behavior were >1 SD above the mean (*n* = 100, 86, and 87 for SF, NSF, and SR, respectively), and more fearful dogs were <1 SD below the mean (*n* = 89, 110, and 73 for SF, NSF, and SR, respectively). We used two different approaches to calculate the GRS: (1) a simple risk allele count method (count GRS, cGRS) and (2) a weighted method (weighted GRS, wGRS). For cGRS, we considered the minor allele (B) as the risk allele and major allele (A) as the protective allele. BB genotypes were counted as 2, AB as 1, and AA as 0. For an SNP marker with a negative effect size, the risk allele homozygotes were counted as 0. Finally, the number of risk alleles was summed up for each individual dog. For wGRS, each risk allele is weighted by the effect size for that allele, giving a linear combination of the risk alleles (weighted by effect size as coefficients). Density plots of risk alleles and weighted risk scores were compared between more fearful and less fearful dogs using the *sm.density.compare* function of R in the *sm* package [38]. The PROC GLM of SAS v.9.4 (SAS Inst., Cary, NC, USA) was used to determine the differences between more fearful and less fearful dogs for risk alleles and weighted risk scores. The Tukey–Kramer method was applied to control for experiment-wise error. *p* < 0.05 was considered significant.

## 3. Results

### 3.1. Inbreeding Coefficient Comparisons Between CB Cohort and Breed Background

A total of 16 breeds met the required sample size of 10 dogs from both the CB cohort and the breed background population, and the sample numbers are included in Table 1, along with the calculated inbreeding coefficient means and variances for each population. *T*-test *p*-values demonstrated that the dogs from CB kennels had significantly lower IBCs than same-breed dogs from the breed background population for all but three breeds. In the three breeds where the difference was not significant (i.e., Cavalier King Charles Spaniel, French Bulldog, and Pomeranian), the CB population still had a lower IBC compared to the general population.

### 3.2. Heritability Estimations of Behavioral Phenotypes in CB Cohort

The genetic contribution (heritability) to each behavioral phenotype (SF, NSF, and SR) was estimated using multiple programs and is summarized in Table 2. SF and NSF were moderately heritable, and both were much more heritable than startle response. The latter appears to have a very strong environmental influence, as the heritability was very low. Additional details specific to each phenotype are described further below.

### 3.3. GWAS of Behavioral Phenotypes in CB Cohort, with Positional Candidate Genes

#### 3.3.1. Social Fear (SF)

**Figure 1:**
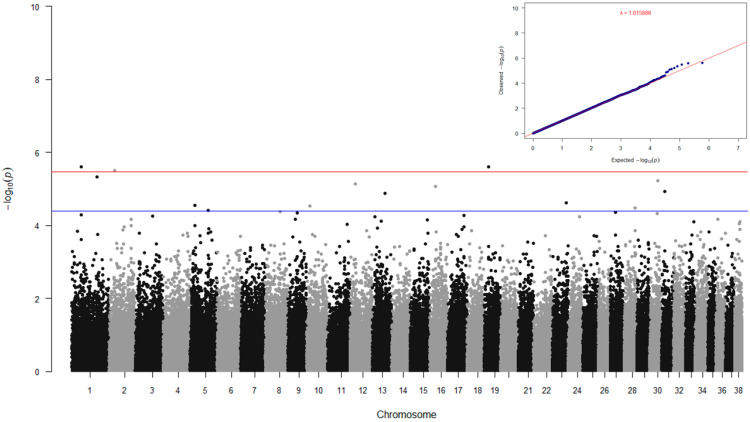
Manhattan plot and QQ plot for social fear in a CB cohort. Red and blue lines in the Manhattan plot indicate the Wald test *p*-values of 3.54 × 10^−6^ (suggestive threshold) and 4.00 × 10^−5^, respectively. The genomic inflation factor (lambda) for the QQ plot is 1.016.

Fixed effects included in the final generalized linear model were facility (*p* < 0.0001), principal component 1 based on the variance-standardized genetic relationship matrix (*p* < 0.0001, correcting for breed population structure), body size (*p* < 0.0001), and sex (*p* < 0.0268); these fixed effects adjusted the observed SF behavioral phenotype. The heritability of SF after phenotype adjustment ranged from 0.267 to 0.324, which indicates SF is a moderately heritable trait (Table 2). We next performed GWAS for adjusted SF using a univariate linear mixed model in GEMMA, correcting for kinship using a relatedness matrix covariate. The GWAS for SF (Figure 1) identified twelve significantly associated variants across 12 chromosomes above the threshold of Wald test *p*-value of 4.00 × 10^−5^ (blue line on Figure 1 Manhattan plot). These twelve markers are listed in Table 3, along with nearby positional candidate genes of interest. For brevity, the positional candidate genes will be highlighted here in terms of their functions; their connections to behavioral phenotypes will be described further in the Discussion section.

One CFA1 (*Canis familiaris)* variant (rs21883975) was located 426.6 kb downstream of the *Riboflavin Kinase* (*RFK*) gene. The RFK protein catalyzes riboflavin’s phosphorylation to form flavin mononucleotide (FMN) in the presence of ATP and Mg^2+^ or other divalent ions [39]. The FMN is a redox cofactor in energy production and is essential to cell survival [40]. One CFA5 variant (rs24230782) was 294.5 kb upstream of the *Nephrocystin 4* (*NPHP4*) gene, which encodes the NPHP4 protein. This protein is essential for the development and function of renal tubules [41] and also plays a role in facilitating ciliary sensory signal transduction [42]. The CFA10 variant (AX-167584753) was 267.9 kb upstream of the *Protein Tyrosine Phosphatase Receptor Type B* (*PTPRB*) gene. The extracellular region of the PTP protein contains multiple fibronectin type III repeats that interact with neuronal receptors and cell adhesion molecules, like contactin and tenascin C [41]. The CFA13 variant (AX-168016904) was 261.9 kb upstream of the *γ-Aminobutyric Acid Type A Receptor Subunit Alpha2* (*GABRA2*) gene. GABA (γ-aminobutyric acid) is the primary inhibitory neurotransmitter in the mammalian central nervous system (CNS) [41]. *GABRA2* encodes a GABA-A receptor subunit, which is activated by GABA [43]. GABA-A receptors, together with GABA-B receptors, mediate the inhibitory effects of GABA in the CNS [44]. A CFA28 variant was located within 12.1 kb upstream of the *neuropeptide S* (*NPS*) gene. This gene encodes a 20 amino acid neuropeptide in the mammalian brain that binds to its agonist, G-protein-coupled receptor (NPS receptor, NPSR1) protein, to increase free intracellular calcium levels and cyclic adenosine monophosphate accumulation [45]. Finally, the CFA30 variant was 138.6 kb downstream of the *Vacuolar Protein Sorting-associated 13 Homolog C* (*VPS13C*) gene. *VPS13C* encodes a protein that regulates mitochondrial function through the endolysosomal pathway in neurons [46].

#### 3.3.2. Non-Social Fear (NSF)

**Figure 2:**
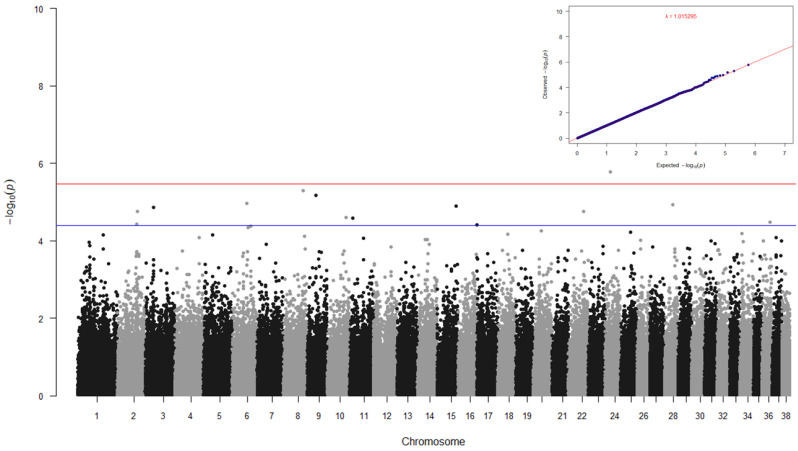
Manhattan plot and QQ plot for non-social fear in a CB cohort. Red and blue lines in the Manhattan plot indicate the Wald test *p*-values of 3.54 × 10^−6^ (suggestive threshold) and 4.00 × 10^−5^, respectively. The genomic inflation factor (lambda) for the QQ plot is 1.015.

None of the fixed effects (facility, principal components 1 and 2, body size, sex, or age) were significant for NSF (*p* ≥ 0.0985) in this CB cohort. The heritability of NSF obtained using several programs ranged from 0.287 to 0.354 (Table 2), indicating that NSF is also a moderately heritable trait. GWAS of NSF was performed using a univariate linear mixed model in GEMMA, where a relatedness matrix was again used to control for kinship. The variants surpassing the Wald test *p*-value < 4.00 × 10^−5^ (*n* = 12, Figure 2), with their respective positional candidate genes of interest, are shown in Table 4.

A CFA2 variant was located 300 kb downstream of the *C-X3-C Motif Chemokine Ligand 1* (*CX3CL1*) gene and 133.3 kb upstream of the *Nucleoporin 93* (*NUP93*) gene. The *CX3CL1* gene encodes Fractalkine, a chemokine ligand 1 expressed by neurons in the CNS. This chemokine regulates microglia activation through its receptor, CX3CR1, in response to brain injury or inflammation [47]. The product of the *NUP93* gene is a target of caspase cysteine proteases, which have a vital role in programmed cell death or apoptosis [48]. An intronic variant was identified in the *Ras Protein Specific Guanine Nucleotide Releasing Factor 2* (*RASGRF2*) gene on CFA3. This gene encodes a calcium-regulated nucleotide exchange factor that activates Ras and Ras-related protein, RAC1, by switching the inactive GDP-bound state to the active GTP-bound state [41]. Both Ras and RAC1 proteins significantly influence brain functions and neurodevelopment, including neurogenesis, differentiation, synaptic plasticity, neuronal migration, axon development, and memory function via the regulation of actin dynamics in neurons [49,50]. The CFA9 variant was 304.9 kb downstream of the *Malignant brain tumor domain containing 1* (*MBTD1*) gene. This gene encodes a protein of the polycomb group family that is associated with tumorigenesis [51]. The variant on CFA15 was located 199 kb downstream of the *Follistatin like 5* (*FSTL5*) gene. This gene encodes an extracellular matrix secretory protein, which has a similar molecular structure like follistatin [52]. The FST-like family proteins regulate organ development, cell proliferation, migration, and differentiation [52]. A CFA17 variant was 97.1 kb upstream from the *Peroxidasin* (*PXDN*) gene, which encodes a heme-containing peroxidase involved in extracellular matrix formation [41]. A CFA24 variant was upstream from three different protein-coding genes [*Prion Protein* (*PRNP*), *Prion Like Protein Doppel* (*PRND*), and *Solute Carrier Family 23 Member 2* (*SLC23A2*)]. Prion protein is active in the brain and several other tissues [53]. This protein plays a significant role in several biological processes, such as neuritogenesis, neuronal homeostasis, cell signaling, and cell adhesion, and has a protective function against stress [53]. In dogs, *PRND* is located 20 kb upstream from the gene encoding prion protein. The *PRND* (Doppel) product may be neurotoxic when overexpressed, leading to neurological disorders. However, prion protein counters and prevents Doppel-induced neurotoxicity because of their antagonistic interactions [54]. *SLC23A2* encodes sodium-dependent vitamin C transporter 2 (SVCT2), which regulates vitamin C absorption into the body and its distribution into different organs [41]. An earlier study reported hypomyelination and reduced extracellular matrix components in the peripheral nerve of SVCT2 deficient mice [55]. The CFA28 variant was 152.4 kb upstream of the *adrenergic β-1 receptor* (*ADRB1*) gene. *ADRB1* encodes an adrenergic receptor, a G-protein-coupled receptor that regulates the physiological effect of epinephrine and norepinephrine [41]. These chemicals act as neurotransmitters and hormones in the body and play an essential role in panic, excitement, anxiety, stress, and aggression [56]. Finally, an intronic CFA36 variant was identified in the *Phosphodiesterase 1A* (*PDE1A*) gene. This gene is highly expressed in the cerebral cortex and hippocampus in the brain [57]. The PDE1A protein catalyzes the hydrolysis of cyclic adenosine monophosphate (cAMP) and cyclic guanosine monophosphate (cGMP); cAMP plays a crucial role as a second messenger in several neuronal functions, including growth cone motility, neuronal metabolism, axon extension in vitro, neuroprotection, and survival in vivo [58].

#### 3.3.3. Startle Response (SR)

**Figure 3:**
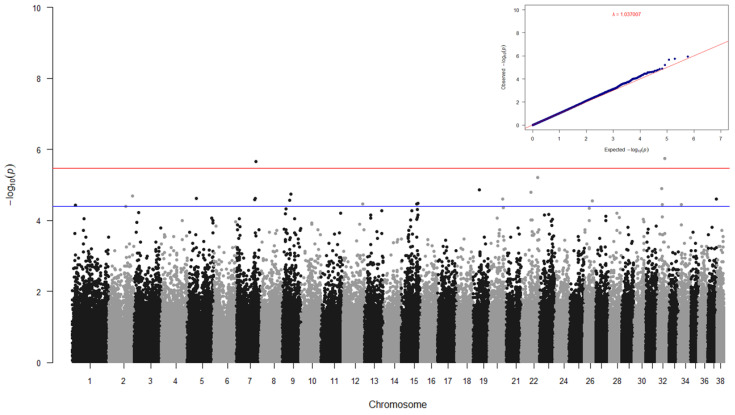
Manhattan plot and QQ plot for startle response in a cohort of dogs from CB facilities. Red and blue lines in the Manhattan plot indicate the Wald test *p*-values of 3.54 × 10^−6^ (suggestive threshold) and 4.00 × 10^−5^, respectively. The genomic inflation factor (lambda) for the QQ plot is 1.037.

The SR phenotypic values were adjusted using the significant fixed effects, which included body size (*p* < 0.0137), age (*p* < 0.0013), and sex (*p* < 0.0442) of dogs. Neither facility nor any principal components were significant for SR (*p* ≥ 0.1103). Heritability of SR was calculated in multiple programs, with heritability ranging from 0.042 to 0.105, indicating SR is a lowly heritable trait (Table 2). GWAS was performed with adjusted phenotypes using a univariate linear mixed model in GEMMA; relationship stratification was corrected for using a kinship matrix in the model. The SR GWAS findings are shown in Figure 3, and eighteen associated variants (on 13 chromosomes; Wald test *p*-value < 4.00 × 10^−5^) are shown in Table 5, together with their respective positional candidate genes of interest.

The CFA2 variant was 244.4 kb downstream of the *aldehyde dehydrogenase 4 family member A1* (*ALDH4A1*) gene. Polymorphisms in *ALDH4A1* are associated with metabolic diseases, mainly characterized by neurological manifestations [59]. A CFA5 variant was located 61.5 kb downstream of the *KIAA0753* gene. The product of this gene regulates ciliogenesis and cilia maintenance [41]. On CFA19, one variant was 191.4 kb downstream of the *Pleckstrin homology domain containing B2* (*PLEKHB2*) gene. The PLEKHB2 protein enables phosphatidylinositol-3,4,5-trisphosphate binding activity and regulates cell differentiation [41]. One CFA26 variant (rs23330578) was 338.6 kb and 223.6 kb upstream of the *Adenosine A2A receptor* (*ADORA2A*) and *Sperm Antigen With Calponin Homology And Coiled-Coil Domains 1 Like* (*SPECC1L*) genes, respectively. The *ADORA2A* gene encodes a G-protein-coupled receptor that regulates myocardial oxygen consumption, coronary blood flow, and CNS neurotransmitters [60]. The *SPECC1L* protein plays a role in the stability of the microtubules, actin cytoskeleton reorganization, cell migration, and adhesion [61]. Finally, a CFA37 variant was located 183.2 kb upstream of the *WD repeat and FYVE domain containing 1* (*WDFY1*) gene. This gene encodes a phosphatidylinositol 3-phosphate binding protein, which positively regulates Toll-like receptors (TLR) 3 and 4 signaling pathways by recruiting TIR-domain-containing adapter-inducing interferon- β to these receptors [62].

### 3.4. Genetic Risk Scores of Behavioral Phenotypes in a CB Cohort

**Figure 4:**
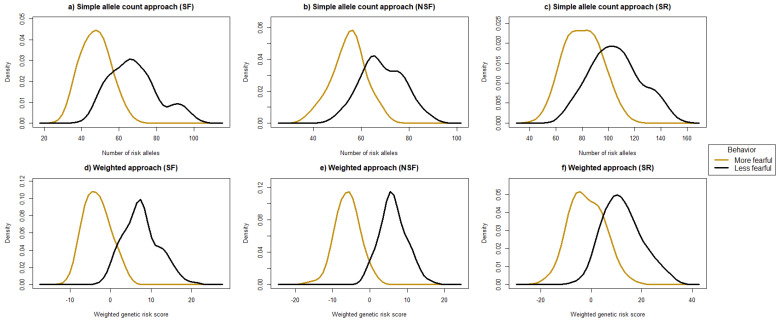
Density plots comparing risk allele counts (cGRS) and weighted genetic risk scores (wGRS) between more fearful and less fearful dogs in a CB cohort: (**a**–**c**) density plots of cGRS (simple risk allele counts) for SF, NSF, and SR, respectively; and (**d**–**f**) density plots of wGRS (weighted risk scores) for SF, NSF, and SR, respectively. Note that the x- and y-axes are not identical for each panel.

In order to obtain discrete categories of dogs for comparison purposes, dogs were binned as less fearful, intermediate, and more fearful. First, categorizing dogs into the two more extreme bins (less fearful and more fearful in response to test stimuli), defined as >1 SD above or below the mean, retained a subset of dogs from the total cohort (*n* = 575) for each of the three phenotypes. For SF, 189 dogs were retained (100 less fearful and 89 more fearful, as defined by >1 SD above or below the mean); for NSF, 196 dogs were retained (86 less fearful and 110 more fearful, as defined by >1 SD above or below the mean); and, for SR, 160 dogs were retained (87 less fearful and 73 more fearful, as defined by >1 SD above or below the mean). The distribution of risk alleles (cGRS) or weighted risk scores (wGRS) in more fearful and less fearful dogs is shown in Figure 4. In both approaches, there is a clear separation of risk alleles and weighted risk scores between more fearful and less fearful dogs for all behavioral phenotypes. Moreover, the average weighted risk score and risk allele numbers were significantly higher in less fearful dogs than in more fearful dogs (Table 6).

Finally, we categorized all studied dogs (*n* = 575) into three bins (more fearful, intermediate, and less fearful) for all three behavioral traits by retaining less fearful dogs (behavioral phenotypes > 1 SD above the mean), more fearful dogs (phenotypes > 1 SD below the mean), and adding the third category of all dogs between these extremes (the intermediate group). Density plots using these groups of dogs reveal intermediate dogs falling between the other two groups (Figure S1). Similarly, the average cGRS and wGRS of intermediate dogs differed significantly from the other two categories except for cGRS for SR (Table S5).

## 4. Discussion

This is the first canine behavioral genetics study of a cohort of dogs from CB facilities, in which inbreeding coefficients, heritability, GWAS, and genetic risk scores were analyzed. Our data indicated that a population of dogs from CB facilities had either non-statistically different, or significantly lower levels of inbreeding (IBCs) compared to the breed background population for all breeds that met the inclusion criteria. There are, however, limitations to these data. First, the inclusion criteria required at least ten dogs from the same breed in both datasets; although representative, ten dogs cannot truly capture the inbreeding across an entire breed, nor can it truly represent a full picture of either population. The results should, therefore, be confirmed by using an increased sample size in future studies. In addition, there may also be ascertainment bias between the two populations used to calculate IBCs. On the one hand, kennels contributing to the CB cohort varied in size, management practices, and housing environment, and were recruited specifically based on facility (the fact that they were CB kennels). On the other hand, all dogs used for the breed background population had SNP data previously generated for other research projects (ours, or those made publicly available, typically investigating inherited trait or disease phenotypes), and were typically recruited based on phenotype, and are used here opportunistically. Despite these limitations, the results suggest that dogs from this sampling of CB facilities were subject to less linebreeding or inbreeding than dogs from the breed background (typically the show/hobby) breeding population. It is well-known that increased inbreeding leads to decreased genetic diversity and increased dissemination of deleterious alleles [63]; the present study indicates that dogs from CB facilities have a broader gene pool and, if so, may experience a degree of genetic advantage over dogs of the same breed from the breed background population. This may be a reflection of breeder priorities; i.e., some commercial breeders deliberately prioritize genetic diversity considerations, and, therefore, make efforts to bring varied, within-breed genetics into their lines.

Next, we calculated the heritability of the three behavioral phenotypes, SF, NSF, and SR using genome-wide SNP data. We began with SF, which has both genetic and learned components. In the present study, SF represented dogs’ responses to an unfamiliar person moving increasingly closer to them to the point of attempting to interact with them in their kennels, and was found to be moderately heritable (estimates range from 0.267 to 0.324). These results are very similar to heritability estimates reported in earlier studies, although such studies were pedigree-based and did not utilize genotype data. For example, Persson et al. [64] found heritabilities of 0.32 and 0.23 for human-directed social behaviors in research Beagles. Arvelius et al. [65] reported a heritability of 0.25 for stranger-directed fear in Rough Collies. The heritability for dog-directed aggression or fear was 0.23 in 14 Swedish dog breeds [66], 0.00 to 0.18 in puppies (6 and 12 months of age) of three guide dog breeds (Labrador Retrievers, Golden Retrievers, and German Shepherd dogs) [67], and 0.12 in Rough Collies [65]. Chronic or extreme manifestations of SF may significantly reduce the dogs’ quality of life whilst at the kennel as well as in the future transition to a family home after their retirement from breeding [4,22]. Knowing the heritability of traits such as SF paves the way to inform best breeding practices, by raising awareness about the implications of selecting for affiliative behavior toward people and other animals.

The heritability of non-social fear, the most common behavioral problem in adult dogs [68], is determined by both genetic and environmental factors. An earlier study identified several environmental factors affecting NSF in dogs, such as socialization, owners’ dog experience, activities/training, the presence or absence of other dogs in the family, daily exercise, urban environment, and family size [68]. Dogs experiencing NSF show fearfulness (extreme agitation, panting, or attempting to escape or hide) towards stimuli, such as novel objects, sudden or loud noises, novel situations, and surfaces and heights [68,69]. In the current study, NSF represented dogs’ responses to inanimate objects placed inside their kennels (i.e., mat, leash, cone, or dog statue). A moderate heritability estimate was observed for NSF in our study (estimates range from 0.287 to 0.354). This is similar to results reported in previous studies, where heritability estimates for NSF were 0.19 in 14 different Swedish dog breeds [66], 0.01 to 0.27 in puppies of three guide dog breeds (Labrador Retrievers, Golden Retrievers, and German Shepherd dogs) [67], and 0.36 in Rough Collies [65]. Clearly, NSF in dogs (in keeping with all behaviors) is controlled by a number of genes as well as the environment.

The third behavioral phenotype described in this study is startle response, which was represented in our study by dogs’ responses to an opening and closing umbrella. In general, startle response is characterized by a dog responding to normal stimuli with an abnormal level of intensity, potentially also with a prolonged duration and recovery [70,71]; as such, startle response comprises a combination of factors, including the size of the response, the threshold for what it takes to elicit the response, and the time to recovery. Startle response may manifest as excessive barking around another dog, a non-threatening stranger, a passing vehicle, or any other stimuli causing a significant overreaction [72]. Such dogs typically exhibit alertness, restlessness, and vocalization, and can also demonstrate systemic effects (such as vomiting, urination, or defecation), and stereotypic behaviors [73]. Heritability for SR in the present study (estimates range from 0.042 to 0.105) reveals that this is a lowly heritable trait, particularly in comparison to SF and NSF. Besides genetic contributions, a high startle response or reactive behavior in dogs can be caused by a lack of socialization, a lack of training, a frightening experience, an extremely stimulating environment, or a combination of these [74]. While many genes might have a negligible effect, given that startle response is a sub-section of fear, it is clear the environment(s) significantly affects this behavioral characteristic.

Our next step was to conduct GWAS for the three behavioral phenotypes. Beginning with SF as a continuous phenotype, GWAS highlighted some potential neuronal candidate genes. The first candidate gene highlighted by the GWAS is riboflavin kinase (*RFK*). MacLean et al. [15] reported an association of the *RFK* gene with threatening or aggressive responses of dogs toward other dogs. The next identified candidate gene was *NPHP4*, which was likewise identified in the MacLean et al. study [15] as linked with aggressive or threatening responses of dogs to other familiar dogs in the same household. *PTPRB,* on CFA10, encodes a chondroitin sulfate proteoglycan, which plays a vital role in neural morphogenesis and axon guidance mechanisms [75]. This gene has similarly been previously associated with dog behaviors: MacLean et al. [15] suggested the involvement of the *PTPRB* gene with aggressive responses of dogs toward owners or other household members when challenged, handled, stared at, stepped over, or approached while in possession of food or objects. The PTPRB protein was also associated with a neuronal disorder named pilocarpine-induced epilepsy in rats [75]. Another candidate gene highlighted by the SF GWAS is *GABRA2*. Many studies have indicated GABRA2 is involved in anxiety, stress response, and depression, and it is also thought to have a role in neurological disorders such as early-onset epilepsy, early-onset epileptic encephalopathy, and schizophrenia in humans [43,76,77,78,79]. The neuropeptide S gene (*NPS*) has been linked to anxiety-related disorders, fear behavior, and panic disorder in humans [80]. Lastly for SF, *VPS13C* was reported to be associated with a complex neurodegenerative disorder called autosomal recessive early-onset Parkinson disease 23 [81]. Social anxiety, depression, social avoidance, or phobias involving fear or embarrassment are characteristic symptoms of Parkinson’s disease [82,83,84]. Several studies have already been conducted to elucidate the genetic background of SF-related behavioral traits in dogs, including stranger fear, dog aggression, dog rivalry, dog fear, owner aggression, stranger aggression, and human-directed aggression [13,14,15,17,85,86]. Our study identified three loci (*RFK*, *NPHP4*, and *PTPRB*) for SF, which were previously identified by MacLean et al. [15], who reported an association of those loci with dog aggression, dog rivalry, and owner aggression. It is worth noting that extreme fearful or aggressive reactions in the current tested population were extremely rare (<1%) [20]. The GWAS results should not be interpreted as the genetic characterization of population phenotype; rather, these results indicate that, similar to other pet dog populations studied, the associations found between social fear responses and specific genetic loci are partially confirmed in this cohort of dogs from CB facilities. Previous studies, however, identified several other candidate genes associated with SF-related behavioral traits, which differed from our present study [13,14,15,17,85,86]. These differences in association findings could be due to the different populations, the inclusion of different dog breeds, or owner-completed C-BARQ datasets used in the previous studies compared to the direct observational scoring used in the present study.

The NSF GWAS of the present cohort from CB kennels suggested eleven genes in eight loci. The first GWAS locus was near two genes: *CX3CL1* and *NUP93*. The CX3CL1 protein signals by binding with its receptor CX3CR1 and regulates microglial activation in response to brain damage or inflammation [47]. Any disruptions in the CX3CL1/CX3CR1 signaling pathway may result in the development of neurodegenerative diseases [47]. MacLean et al. [15] reported an association of the *CX3CR1* gene with excitability in dogs. The arrival of visitors or owner(s) coming to the dog’s home or arousal events during playtime, such as loud voices, fast movements, and toys, may trigger excitability in dogs [87]. The same study by MacLean et al. [15] likewise found an association between the *NUP93* gene and attachment/attention-seeking in dogs. Attention-seeking behavior is considered an early sign of separation anxiety in dogs [88]. The GWAS also revealed a candidate gene on CFA3, *RASGRF2*, which was previously associated with fearful or wary responses to potentially painful or uncomfortable procedures, including bathing, grooming, nail clipping, and veterinary examinations [15]. The next candidate gene identified by GWAS was *MBTD1*. This gene was associated with fearful or wary responses of dogs towards unfamiliar people [15]. Another interesting GWAS result was the candidate gene *FSTL5*, which codes for a secretory glycoprotein that is an important paralog of the *FSTL4* gene. Both MacLean et al. [15] and Zapata et al. [17] found an association between the *FSTL4* gene and NSF in dogs, suggesting that this gene family has major involvement in NSF. The current study next identified PXDN, an important paralog of the *PXDNL* gene. MacLean et al. [15] reported an association of the PXDNL gene with excitability in dogs. Three genes (*PRNP*, *PRND*, and *SLC23A2*) are near one another on an identified CFA24 locus. Human *PRND* and *PRNP* share a 24% coding sequence identity [89]. Previous studies reported associations of these two genes (*PRND* and *PRNP*) with the fatal neurodegenerative condition Creutzfeldt–Jakob disease in humans [90,91,92,93]. Patients with Creutzfeldt–Jakob disease often show symptoms of anxiety, fear, aggressive behavior, and depression as a result of the disease process [94]. The *PRNP* gene is also thought to have a role in Huntington-disease-like neurodegenerative disorder in humans [95], which is characterized by behavioral and psychiatric symptoms, including anxiety, depression, aggression, and irritability [96]. An association of the *SLC23A2* gene with separation anxiety in dogs was previously reported [15], dovetailing with the current findings. Taken together, the parallels between our study and previous work in both humans and dogs certainly make this CFA24 locus worth further investigation in dogs with NSF. Next, the G-protein-coupled receptor gene *ADRB1* was also highlighted in the present study, and *ADRB1* gene mutations have been associated with anxiety-related behaviors like social phobia and agoraphobia (extreme and irrational fear of being unable to escape a difficult or embarrassing situation) in humans [97]. Finally, our study identified a locus in a *PDE1A* gene intron, and this gene, likewise, has previously been associated with NSF in dogs [15]. Since the PDE1A protein regulates the hydrolysis of cAMP, which plays a second messenger role in several neuronal functions [58], mutations in *PDE1A* may be involved in neurological differences. In summary, for NSF, we identified two behavioral loci that were previously associated with social anxiety in humans or stranger fear in dogs [15,97], which strongly indicates that common genetic underpinnings could control both SF and NSF phenotypes. An earlier study also supports this, as they reported eight loci shared between stranger-directed fear and NSF in dogs [17]. Taken together, the consistency of genetic loci across dog populations, in both our CB cohort and canine cohorts in other studies, serves to highlight the importance of these particular loci, which should be investigated more thoroughly in the future. However, most of the candidate genes associated with canine NSF in previous studies differed from ours [15,16,17,86]. This discrepancy in association studies likely reflects the genetic complexity of behavioral phenotypes, and could also be due to dog breed type differences. It seems less likely, though, that these discrepant loci are a result of facility affect, given the major overlap that was observed.

Despite its low heritability, our GWAS did identify putative candidate genes for the SR score of dogs in CB facilities. Once again, several identified loci overlap with previously reported behavioral loci from other dog populations. For example, the *KIAA0753* gene has been associated with a fearful or wary response of dogs to unfamiliar dogs [15]. Mutations in this gene are also associated with Joubert syndrome in humans [98], a condition characterized by hyperactivity, and aggressiveness, among many other clinical symptoms [99]. Our SR GWAS also highlighted the genes *PLEKHB2*, *SPECC1L*, and *WDFY1*, which have ties to previous behavioral genetic work in dogs. *PLEKHB2* was previously associated with aggressive responses of dogs toward owners or other household members [15], and the same group reported an association of the *SPECC1L* gene with attachment/attention-seeking behavior in dogs [15]. *WDFY1* is an essential paralogous gene of *WDFY2*, the latter of which was likewise reported in the same study [15] as being associated with aggressive responses of dogs toward other dogs. Finally, two more candidate genes were identified: (1) *ALDH4A1*, which has been linked to a rare autosomal recessive human disorder, Hyperprolinemia type II, with the phenotypes of irritability and aggressive behavior [100]; and (2) *ADORA2A*, which has been previously associated with panic disorder and anxious personality in humans [101]. Taken together, these results serve to reinforce the complexity of the SR phenotype. No previous studies have specifically studied genetic associations with SR scores in dogs, although two earlier studies reported several candidate regions associated with excitability, which is a similar and related phenotype, in dogs [15,86]. However, none of the reported genes specifically for “excitability” overlapped with those observed for SR in our study. Since there are some genes that did overlap between our study (SR phenotype) and others (different behavioral phenotypes), it seems reasonable to consider these loci as underlying behavior more generally, rather than being specific to SR.

As a last modality, we calculated genetic risk scores using two different methods. Both approaches suggested that the number of risk alleles is significantly associated with desirable dog behavior, i.e., minimum or no fearfulness. Dogs with higher genetic risk scores were less fearful compared those with low genetic risk scores. If this difference is sustained across future studies with even more dogs, this suggests that selection pressure could be applied to produce less fearful dogs, although it would take additional resources, including SNP genotyping of the dogs and substantial data analysis. While most of the SNP markers used in this analysis were non-significant, they still demonstrate insights into the allelic effects controlling dog behavior.

Future work could include multivariate analysis (for example, trivariate analysis with SF, NSF, and SR) of these same data to examine the relationships between these traits and their SNPs. This could begin to tease out how selecting for or against one of these phenotypes might positively or negatively affect the others.

The findings from this study cannot be universally assumed to apply to all CB cohorts, because some kennels follow additional/voluntary welfare guidelines, together with different management practices such as socialization and early handling, and others do not; however, the genetic loci that have now been consistently identified for behavioral traits across different populations supports the repeatability of these findings. For non-Mendelian traits in a GWAS study, a much greater sample size is required to achieve adequate statistical power [102]. While the present study has a canine cohort of hundreds, for genetically complex traits such as behavior, investigations would likely benefit from even larger sample sizes, especially if the study is multi-breed such as ours.

Our study confirms several canine genetic behavioral loci observed in previous studies, which strongly suggests these loci are worth further investigation. We also demonstrate the high genetic similarities between genetic behavior phenotypes in this CB cohort and other canine populations used in previous studies. Calculation of IBCs indicated that dogs from CB facilities are less inbred than dogs originating from other breeding sources; this begins to dispel the assumptions about the genetic health of CB populations. Heritability was higher for social fear and non-social fear when compared to startle response. In addition, our study revealed higher risk allele and weighted risk scores in less fearful dogs compared to those that were more fearful dogs, suggesting that these behaviors could be subjected to genetic improvement, given enough resources and time. Future work using more dogs is needed to develop a strong polygenic risk score model, which should eventually allow for predicting desirable behaviors. Taken together, this work also suggests CBs should pay careful attention to the SF and NSF behaviors of dams and sires, selecting those with more affiliative responses. Since puppies from this population are typically sold to the US public, doing so will aid in supporting puppies’ transition to family homes and may help support their welfare whilst in such environments.

## Figures and Tables

**Table 1:** **Mean IBC and variance based on genome-wide SNP data within each population.** The appropriate *t*-test (assuming equal variance or not) was used to calculate a two-tailed *p*-value. Significant *p*-values (*p* < 0.05) are in bold. IBC = inbreeding coefficient. See Table S4 for more specific information, including the number of markers used for each breed.

Breed	Breed Background IBC Mean (Variance)	Commercial Breeding IBC Mean (Variance)	*p*-Value
American Cocker Spaniel	0.072092 (0.008133) *n* = 16	−0.03392 (0.004166) *n* = 34	**1.84 × 10^−5^**
Australian Shepherd	0.05939 (0.009972) *n* = 277	0.005118 (0.004865) *n* = 14	**0.045649**
Bichon Frise	0.045651 (0.006584) *n* = 101	−0.02843 (0.008112) *n* = 36	**1.1 × 10^−5^**
Bullmastiff *	0.030963 (0.007931) *n* = 43	−0.00998 (0.002771) *n* = 15	**0.039**
Cavalier King Charles Spaniel	0.045081 (0.007391) *n* = 254	0.019761 (0.00693) *n* = 44	0.071031
French Bulldog *	0.020821 (0.002795) *n* = 65	−0.00506 (0.007024) *n* = 22	0.185136
Golden Retriever	0.055963 (0.010229) *n* = 471	0.003179 (0.006466) *n* = 22	**0.016242**
Great Dane *	0.049074 (0.013713) *n* = 97	−0.02324 (0.001514) *n* = 14	**2.65 × 10^−5^**
Labrador Retriever	0.041517 (0.006761) *n* = 1424	0.085459 (0.007521) *n* = 25	**0.008221**
Miniature Schnauzer *	0.063033 (0.024241) *n* = 364	−0.08533 (0.005618) *n* = 20	**3.63 × 10^−9^**
Pomeranian	0.039109 (0.007194) *n* = 51	0.007014 (0.006416) *n* = 15	0.196967
Shetland Sheepdog	0.105334 (0.017431) *n* = 60	−0.19236 (0.016604) *n* = 16	**1.03 × 10^−11^**
Shih Tzu *	0.051114 (0.012371) *n* = 93	−0.014 (0.004515) *n* = 49	**2.75 × 10^−5^**
Siberian Husky	0.049934 (0.007221) *n* = 131	0.010516 (0.004038) *n* = 24	**0.032169**
Toy Poodle *	0.041009 (0.0091) *n* = 30	−0.02076 (0.001406) *n* = 22	**0.002523**
Yorkshire Terrier *	0.052383 (0.014324) *n* = 371	−0.00091 (0.003964) *n* = 17	**0.003828**

* Significantly different variances based on F-test.

**Table 2:** SNP-based heritability of behavioral phenotypes in multiple programs.

Behavioral Traits	BLUPF90		GEMMA	GCTA
	AIREMLF90 (h^2^ ± SD)	GIBBSXF90 (h^2^ ± SD)	LMM (h^2^ ± SD)	REML (h^2^ ± SE)
Social Fear	0.309 ± 0.097	0.324 ± 0.089	0.287 ± 0.089	0.267 ± 0.084
Non-Social Fear	0.325 ± 0.109	0.354 ± 0.111	0.307 ± 0.115	0.287 ± 0.097
Startle Response	0.055 ± 0.057	0.105 ± 0.062	0.052 ± 0.055	0.042 ± 0.045

**Table 3:** SNPs associated with the social fear component of a behavioral assessment in a cohort of dogs from CB facilities.

CFA ^a^	SNP	CanFam3.1 Position	Alleles	MAF ^b^	Wald Test *p*-Value	Gene	Gene Location ^c^
1	rs21882651	30431932	C/A	0.226	2.505 × 10^−6^		
1	rs21883975	82495850	A/C	0.3	4.616 × 10^−6^	RFK	426.6 kb
2	AX-167608769	17205702	T/G	0.116	3.211 × 10^−6^		
5	rs851443574	15540307	G/A	0.474	2.790 × 10^−5^		
5	rs24230782	59519768	C/A	0.3	3.913 × 10^−5^	NPHP4	294.5 kb
10	AX-167584753	11973796	A/G	0.436	2.920 × 10^−5^	PTPRB	267.9 kb
12	AX-167551293	17338027	T/C	0.092	7.389 × 10^−6^		
13	AX-168016904	42106964	G/C	0.397	1.350 × 10^−5^	GABRA2	261.9 kb
16	rs22459993	20044351	C/T	0.086	8.610 × 10^−6^		
19	AX-168004135	14622094	A/G	0.215	2.452 × 10^−6^		
23	rs852203828	46770806	G/T	0.373	2.449 × 10^−5^		
28	AX-168063805	36476408	T/C	0.162	3.257 × 10^−5^	NPS	12.1 kb
30	AX-167529891	26915319	T/C	0.051	6.096 × 10^−6^	VPS13C	138.6 kb
31	rs23701876	9125755	A/G	0.274	1.201 × 10^−5^		

^a^ *Canis familiaris* chromosome. ^b^ Minor allele frequency. ^c^ Distance of SNP from the listed annotated gene.

**Table 4:** SNPs associated with the non-social fear component of a behavioral assessment in a cohort of dogs from CB facilities.

CFA ^a^	SNP	CanFam3.1 Position	Alleles	MAF ^b^	Wald Test *p*-Value	Gene	Gene Location ^c^
2	rs22798017	59332140	C/T	0.126	3.720 × 10^−5^	*CX3CL1* *NUP93*	300.0 kb 133.3 kb
2	rs22789819	61587923	G/A	0.296	1.751 × 10^−5^		
3	rs23566277	26281810	A/C	0.241	1.394 × 10^−5^	*RASGRF2*	Intron
6	rs24320921	46000822	T/A	0.209	1.075 × 10^−5^		
8	AX-167922774	61158313	G/A	0.052	5.173 × 10^−6^		
9	rs24548605	27383075	A/G	0.288	6.608 × 10^−6^	*MBTD1*	304.9 kb
10	rs852164641	57880484	A/G	0.308	2.504 × 10^−5^		
11	AX-168183438	10243813	G/A	0.27	2.617 × 10^−5^		
15	AX-167973632	58869922	G/A	0.176	1.257 × 10^−5^	*FSTL5*	199.0 kb
17	rs22595448	739345	G/C	0.329	3.830 × 10^−5^	*PXDN*	97.1 kb
22	rs23049634	47967963	C/T	0.067	1.773 × 10^−5^		
24	rs8898116	16523939	A/T	0.322	1.700 × 10^−6^	*PRNP* *PRND SLC23A2*	301.7 kb 322.3 kb 50.2 kb
28	AX-167230357	24755828	G/C	0.404	1.174 × 10^−5^	*ADRB1*	152.4 kb
36	rs23965716	25320433	G/T	0.481	3.376 × 10^−5^	*PDE1A*	Intron

^a^ *Canis familiaris* chromosome. ^b^ Minor allele frequency. ^c^ Distance of SNP from the listed annotated gene.

**Table 5:** SNPs associated with the startle response component of a behavioral assessment in a cohort of dogs from CB facilities.

CFA ^a^	SNP	CanFam3.1 Position	Alleles	MAF ^b^	Wald Test *p*-Value	Gene	Gene Location ^c^
1	rs21881391	10309970	C/T	0.354	3.8 × 10^−5^		
2	AX-167764911	79954932	A/G	0.131	2.04 × 10^−5^	*ALDH4A1*	244.4 kb
5	rs24156498	30810425	G/T	0.351	2.42 × 10^−5^	*KIAA0753*	61.5 kb
7	rs24444843	62023278	A/G	0.111	2.66 × 10^−5^		
7	rs851718515	63538570	G/A	0.314	2.4 × 10^−5^		
7	rs24404292	66336325	G/A	0.424	2.17 × 10^−6^		
9	AX-167657840	24670780	G/A	0.257	2.73 × 10^−5^		
9	AX-167350287	28981270	T/C	0.059	1.83 × 10^−5^		
12	AX-167864615	67088312	A/C	0.251	3.46 × 10^−5^		
15	rs22351594	52287707	T/C	0.383	3.44 × 10^−5^		
15	AX-168018831	56041349	G/C	0.17	3.31 × 10^−5^		
19	AX-167734263	21105389	C/A	0.49	1.38 × 10^−5^	*PLEKHB2*	191.4 kb
20	rs852734959	47020493	A/G	0.082	2.5 × 10^−5^		
21	rs22950182	50201135	T/C	0.22	1.18 × 10^−6^		
22	AX-167792016	32725137	T/C	0.101	1.61 × 10^−5^		
22	AX-167851114	55373238	A/G	0.325	6.28 × 10^−6^		
26	rs23330578	27802436	T/C	0.408	2.78 × 10^−5^	*ADORA2A* *SPECC1L*	338.6 kb 223.6 kb
32	rs8560617	14527559	A/G	0.268	1.26 × 10^−5^		
32	AX-168102956	16351940	C/A	0.297	3.58 × 10^−5^		
32	rs23752954	23452265	A/G	0.37	1.77 × 10^−6^		
34	rs9142395	9319761	C/T	0.269	3.58 × 10^−5^		
37	AX-167557734	29260889	A/G	0.102	2.52 × 10^−5^	*WDFY1*	183.2 kb

^a^ *Canis familiaris* chromosome. ^b^ Minor allele frequency. ^c^ Distance of SNP from the listed annotated gene.

**Table 6:** Mean ± SD, median, and range of cGRS and mean ± SD of the wGRS in less fearful and more fearful dogs from a CB cohort.

Phenotype		cGRS (Simple Risk Allele Count Method)			wGRS (Weighted Risk Score Method)	
		Mean ± SD	Median (Range)	*p*-Value	Mean ± SD	*p*-Value
SF	More fearful	47.37 ± 7.26	47 (34–65)	<0.0001	−3.36 ± 3.09	<0.0001
	Less fearful	67.85 ± 12.89	66 (44–99)		7.53 ± 4.33	
NSF	More fearful	54.80 ± 6.77	55 (38–70)	<0.0001	−5.99 ± 3.25	<0.0001
	Less fearful	68.81 ± 8.85	67.5 (50–89)		6.26 ± 3.53	
SR	More fearful	80.49 ± 13.35	81 (53–110)	<0.0001	−1.69 ± 6.67	<0.0001
	Less fearful	105.49 ± 18.99	105 (70–146)		12.46 ± 7.65	

## Data Availability

All data including raw SNP genotypes are uploaded online and available together with the Supplemental Information.

## Supplementary Materials

The following supporting information can be downloaded at: https://www.mdpi.com/article/10.3390/genes15121611/s1, Figure S1: Density plots comparing risk allele counts (cGRS) and weighted genetic risk scores (wGRS) between more fearful, intermediate, and less fearful dogs in a CB cohort; Table S1: List of dog breeds, sex per breed, and total numbers used in the study; Table S2: Description of each step of the reactivity test protocol (adapted from [20]); Table S3: Principal components extracted by the principal components analysis (PCA); Table S4: Specific information for calculation of inbreeding coefficients by breed; Table S5: Mean ± SD, median, and range of cGRS and mean ± SD of the wGRS in more fearful, intermediate, and less fearful dogs from a CB cohort.
