# Supplementary material for: Heritability and Genome-Wide Association Study of Dog Behavioral Phenotypes in a Commercial Breeding Cohort
Source: Genes (Basel). 2024 Dec 17;15(12):1611. doi: 10.3390/genes15121611 (PMC11675989; doi:10.3390/genes15121611)
Supplement: Supplementary file 1 [file genes-15-01611-s001.zip › Supplemental Table S1.pdf]

Supplemental Table S1. List of dog breeds, sex per breed, and total numbers used in the study

Breed/Breed Mix	Sex		Total
	Male	Female	
Alaskan Malamute	2	2	4
American Cocker Spaniel	3	31	34
American Eskimo	0	5	5
Australian Shepherd	4	10	14
Bernese Mountain	1	1	2
Bichon Frise	1	35	36
Boston Terrier	1	4	5
Boxer	0	4	4
Bulldog	0	6	6
Bullmastiff	3	12	15
Cairn Terrier	0	2	2
Cavalier King Charles Spaniel	5	39	44
Chihuahua	0	17	17
Coton de Tulear	0	1	1
Dachshund	5	14	19
French Bulldog	6	16	22
German Shepherd	0	1	1
Golden Retriever	4	18	22
Great Dane	5	9	14
Havanese	7	20	27
Jack Russell Terrier	0	4	4
Labrador Retriever	4	21	25
Lhasa Apso	0	2	2
Maltese	5	17	22
Miniature American Shepherd	1	3	4
Miniature Pinscher	0	2	2
Miniature Poodle	2	4	6
Miniature Schnauzer	5	16	21
Neapolitan Mastiff	2	13	15
Newfoundland	1	0	1
Old English Sheepdog	1	0	1
Ori Pei	0	1	1
Papillion	0	7	7
Pekingese	0	2	2
Pembroke Welsh Corgi	1	2	3
Pomeranian	2	13	15
Pug	0	4	4
Rottweiler	4	5	9
Saint Bernard	2	7	9
Samoyed	1	2	3
Scottish Terrier	0	3	3
Shetland Sheepdog	1	15	16

**Breed/Breed Mix**	**Sex**		**Total**
	**Male**	**Female**	
Shiba Inu	1	4	5
Shih Tzu	7	42	49
Siberian Husky	4	20	24
Silky Terrier	1	3	4
Standard Poodle	2	5	7
Toy Poodle	6	16	22
Yorkshire Terrier	5	12	17
Mixed (Bichon × Poodle)	0	1	1
Mixed (Maltese × Yorkshire Terrier)	0	1	1
Mixed (Pomeranian × Siberian Husky)	1	0	1
Mixed (Shih Tzu × Poodle)	0	2	2
Mixed (Golden Retriever × Doodle)	0	9	9
Mixed (Golden Retriever × Miniature or Toy Poodle)	0	4	4
**TOTALS**	106	509	615
