# Supplementary material for: Heritability and Genome-Wide Association Study of Dog Behavioral Phenotypes in a Commercial Breeding Cohort
Source: Genes (Basel). 2024 Dec 17;15(12):1611. doi: 10.3390/genes15121611 (PMC11675989; doi:10.3390/genes15121611)
Supplement: Supplementary file 1 [file genes-15-01611-s001.zip › Supplemental Table S2.pdf]

Supplemental Table S2. Description of each step of the reactivity test protocol (adapted from[20])

Subtest	Definition
Rubber mat	A rubber mat was positioned on the pen floor with a treat on top. The response of the dog was recorded. The mat was left in the pen for the remainder of the test.
Leash	A slip leash was placed on top of the mat with a treat between it. The response of the dog was recorded. The leash was left in place for the entire test.
Cone	A plastic traffic cone was positioned inside the pen for 30 sec. The response of the dog was recorded.
Problem solving	A treat was placed on the mat and under an upside-down bowl. The dog's success in retrieving the treat was recorded.
Squeaky toy	A plastic squeaky dog toy was squeezed in front of the dog for up to 10 times. The initial and final reaction of the dog to the squeaker was recorded.
Ball toy	A rubber ball was placed on the mat for 30 sec. The response of the dog was recorded.
Dog Statue	A life-like dog statue was positioned inside the pen for 30 sec. The response of the dog was recorded.
Umbrella	An umbrella was opened-closed up to 10 times in front of the dog. The initial and final response of the dog was recorded.
Command	The experimenter called the dog to 'come' up to two times then asked the dog to 'sit' up to two times. The response of the dog was recorded. A treat was offered at the end of each cue regardless of the dog's response.
Loop leash	The experimenter held the leash in an open loop and attempted to slip it over the dog's head. A treat was used to lure the dog inside the loop. The dog could retreat at any time as the loop was never closed around the dog's neck. The response of the dog was recorded.
