# Supplementary material for: Heritability and Genome-Wide Association Study of Dog Behavioral Phenotypes in a Commercial Breeding Cohort
Source: Genes (Basel). 2024 Dec 17;15(12):1611. doi: 10.3390/genes15121611 (PMC11675989; doi:10.3390/genes15121611)
Supplement: Supplementary file 1 [file genes-15-01611-s001.zip › Supplemental Table S3.pdf]

Supplemental Table S3: Principal components extracted by the principal component analysis (PCA). Loadings higher than 0.55 are in **bold**.

	1 Social fear (SF)	2 Food motivation (FM)	3 Non-social fear (NSF)	4 Startle response (SR)
OpenRYG	**.769**	.205	.277	.212
ReachRYG	**.761**	.246	.223	.244
Touch	**.760**	.247	.105	.177
ApproachRYG	**.659**	.165	.382	.082
Command_come	**.616**	.362	.338	.350
Squeaky toy _final	**.602**	.296	.304	.278
Squeaky toy_initial	**.587**	.283	.322	.293
Loop leash	.547	.288	.238	.512
Command_sit_treat	.362	**.778**	.128	.250
Command_come_treat	.409	**.744**	.189	.193
Loop leash_treat	.297	**.692**	.114	.415
Command_sit	.491	**.682**	.182	.248
Leash_treat	-.007	**.645**	.528	.107
Mat_treat	.083	**.644**	.511	.053
Open_Treat	.556	**.620**	.146	.020
Reach_Treat	.598	**.619**	.092	.026
Approach_Treat	.328	**.551**	.296	-.127
Problem solving	.086	.458	.456	.165
Mat	.230	.170	**.777**	.125
Leash	.250	.298	**.774**	.157
Dog statue	.237	.025	**.659**	.282
Cone	.375	.183	**.577**	.350
Ball toy	.321	.244	**.573**	.011
Umbrella_initial	.212	.112	.153	**.809**
Umbrella_final	.227	.130	.230	**.804**

Extraction Method: Principal Component Analysis.

Rotation Method: Varimax with Kaiser Normalization.
