# Supplementary material for: Heritability and Genome-Wide Association Study of Dog Behavioral Phenotypes in a Commercial Breeding Cohort
Source: Genes (Basel). 2024 Dec 17;15(12):1611. doi: 10.3390/genes15121611 (PMC11675989; doi:10.3390/genes15121611)
Supplement: Supplementary file 1 [file genes-15-01611-s001.zip › Supplemental Table S5.pdf]

Table S5. Mean±SD, median, and range of cGRS and Mean±SD of the wGRS in more fearful, intermediate, and less fearful dogs from a CB cohort

Phenotype		cGRS (simple risk allele count method)			wGRS (weighted risk score method)	
		Mean±SD	Median (Range)	*p-value*	Mean±SD	*p-value*
SF	More fearful	47.37±7.26	47 (34 – 65)	<.0001	-3.36±3.09	<.0001
	Intermediate	55.22±10.39	55 (27 – 95)		1.34±3.77	
	Less fearful	67.85±12.89	66 (44 – 99)		7.53±4.33	
NSF	More fearful	54.80±6.77	55 (38 – 70)	<.0001	-5.99±3.25	<.0001
	Intermediate	61.34±9.04	61 (35 – 85)		1.24±3.84	
	Less fearful	68.81±8.85	67.5 (50 – 89)		6.26±3.53	
SR	More fearful	80.49±13.35[b]	81 (53 – 110)		-1.69±6.67	≤.0008
	Intermediate	83.95±15.92[b]	82 (48 – 152)		1.89±7.83	
	Less fearful	105.49±18.99[a]	105 (70 – 146)		12.46±7.65	

[a,b]Means without a common superscript letter differ (*P* < 0.0001)
