# Supplementary figures and images for: Heritability and Genome-Wide Association Study of Dog Behavioral Phenotypes in a Commercial Breeding Cohort
Source: Genes (Basel). 2024 Dec 17;15(12):1611. doi: 10.3390/genes15121611 (PMC11675989; doi:10.3390/genes15121611)

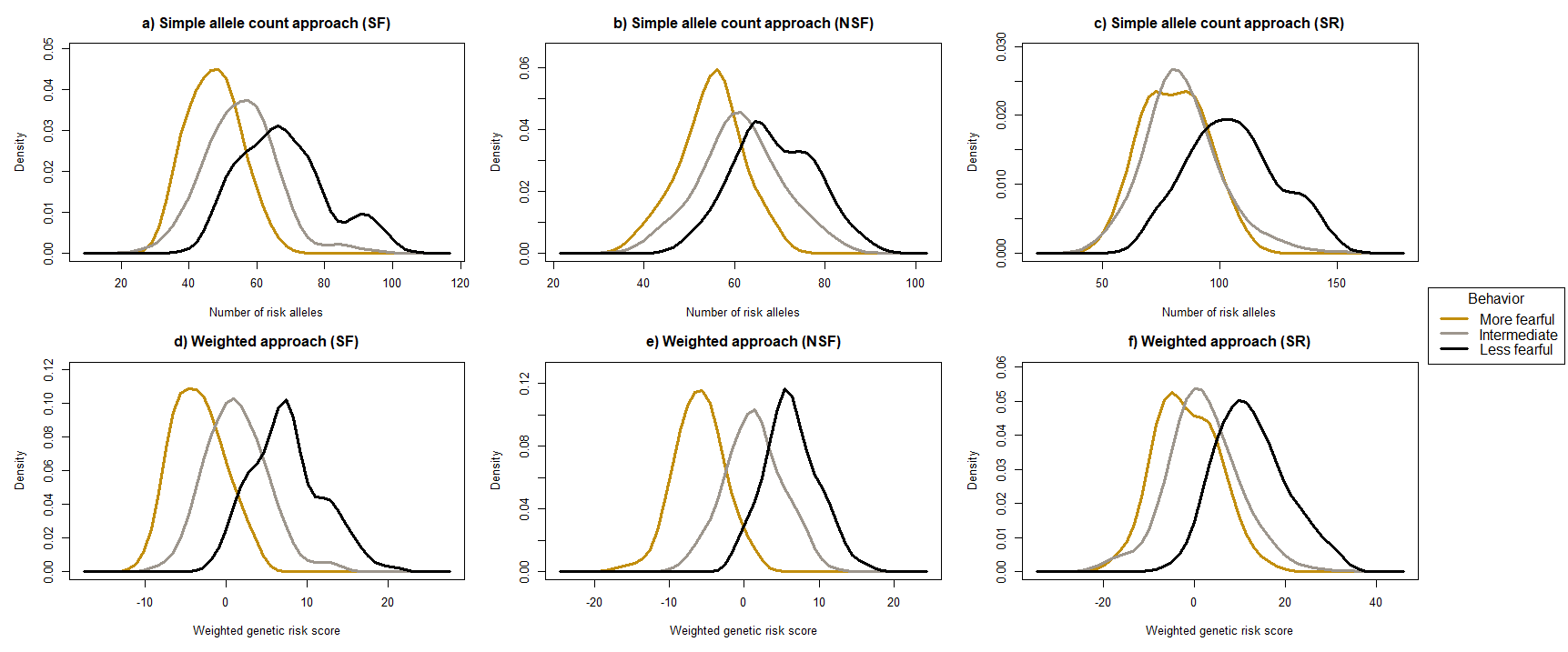

Supplement: Supplementary file 1 [file genes-15-01611-s001.zip › FigureS1_Oct24.tiff]
